# Fiber optic sensors based on hybrid phenyl-silica xerogel films to detect *n*-hexane: determination of the isosteric enthalpy of adsorption

**DOI:** 10.3762/bjnano.8.51

**Published:** 2017-02-21

**Authors:** Jesús C Echeverría, Ignacio Calleja, Paula Moriones, Julián J Garrido

**Affiliations:** 1 Institute for Advanced Materials – Universidad Pública de Navarra, Campus Arrosadía, 31006 Pamplona, Spain; 2 Department of Applied Chemistry – Universidad Pública de Navarra, Campus Arrosadía, 31006 Pamplona, Spain

**Keywords:** fiber optic sensors, isosteric enthalpy of adsorption, *n*-hexane, phenyl-silica, xerogel films

## Abstract

We investigated the response of three fiber optic sensing elements prepared at pH 10 from phenyltriethoxysilane (PhTEOS) and tetraethylsilane (TEOS) mixtures with 30, 40, and 50% PhTEOS in the silicon precursor mixture. The sensing elements are referred to as Ph30, Ph40 and Ph50, respectively. The films were synthesized by the sol–gel method and affixed to the end of optical fibers by the dip-coating technique. Fourier transform infrared spectroscopy, N_2_ adsorption–desorption at 77 K and X-ray diffraction analysis were used to characterize the xerogels. At a given pressure of *n*-hexane, the response of each sensing element decreased with temperature, indicating an exothermic process that confirmed the role of adsorption in the overall performance of the sensing elements. The isosteric adsorption enthalpies were obtained from the calibration curves at different temperatures. The magnitude of the isosteric enthalpy of *n*-hexane increased with the relative response and reached a plateau that stabilized at approximately −31 kJ mol^−1^ for Ph40 and Ph50 and at approximately −37 kJ mol^−1^ for Ph30. This indicates that the adsorbate–adsorbent interaction was dominant at lower relative pressure and condensation of the adsorbate on the mesopores was dominant at higher relative pressure.

## Introduction

Fiber optic chemical sensors (FOCSs) that employ sensitive films for the detection of volatile organic compounds (VOCs) have received considerable attention. FOCSs for VOCs are generally based on indirect sensing schemes, depending on the wavelength, refractive index or fluorescence of an immobilized indicator probe or an optically detectable label that can be monitored [1–6]. Some advantages of FOCSs over electrical methods are immunity to electromagnetic interference and safety while working with flammable and explosive compounds. Although the xerogel films prepared by the sol–gel method are often considered chemically inert, the films and analytes can interact by one or more mechanisms, such as electrostatic, hydrophobic or hydrogen bonding. Films made of hybrid silica materials synthesized by the sol–gel process are well suited for the preparation of sensing elements. These films have chemical and thermal stability, transparency over a wide wavelength range, controlled porous texture that includes a specific surface area and average pore size distribution, and tunable surface chemistry. In the case of fiber optic reflectance sensors (FORSs), these sensitive films vary their optical properties upon interaction with the analyte, thereby resulting in a change in the reflected light. The sensing elements are prepared by immobilizing active films onto the tip of an optical fiber; as a result, the core at one end of the fiber is coated with a thin film.

The sensing mechanism is based on the change in reflected light when VOC molecules are adsorbed on the silica xerogel film covering the tip of the optical fiber, which acts as an optical cavity for which the fiber–xerogel provides the first interface and the xerogel–vapor provides the second interface. The reflectance of this sensing element may be expressed as follows [7–9]:

[1]
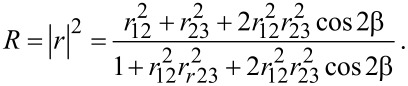


In this equation, the reflectivity at the fiber–xerogel and xerogel–vapor interfaces are expressed by the coefficients *r*_12_ and *r*_23_, respectively, and the parameter β refers to the film thickness and the optical wavelength. In accordance with this equation, any change in the refractive index of the xerogel will lead to a change in the reflectance at the fiber–film interface and the sensor output signal. Furthermore, the reflectance is independent of temperature. The response of fiber optic sensors operating on reflection relies on the complex refractive index of the film and on the adsorption properties, which are related to the porous texture and the interaction or adsorption energy. Molecules cover the external surface, fill the narrow micropores, and condense on meso- and macropores, depending on the relative pressure of the analyte. However, diffusion should also be taken into consideration.

The ability of porous silica and organically modified porous silica films to detect the presence of VOCs under different measurement conditions has been investigated [10–14]. The porous texture and the surface chemistry determine the response. Silanol groups on the surface of the xerogel, which act as weak acids, may interact with molecules that contain lone pairs of electrons, such as acetone; π-electrons, such as toluene; or hydroxyl groups, such as alcohols. The sensing element sensitivity is lower when the xerogel is synthesized from TEOS at pH 10 rather than at pH 4.5.

The effect of temperature on the sensitivity to VOCs has received scarce attention. Adsorption is a spontaneous and exothermic process involving a decrease in the total free energy of the system [15–16]. When a vapor molecule is adsorbed on a surface, this molecule changes from three to two degrees of translational freedom and, as a result, it loses translational entropy. The aim of this study was to assess the effect of temperature on the sensitivity of hybrid phenyl-silica films to *n*-hexane and to determine the variation of the isosteric enthalpy of adsorption.

## Materials and Methods

### Optical fibers and preparation of xerogel films

Multimode optical fibers with a graded refractive index were chosen with core and cladding diameters of 62.5 and 125 µm, respectively (Telnet, Zaragoza, Spain). The effective refractive index was 1.497 at 850 nm. The fibers were first cut and peeled with a stripper (Millar, Cronwell, CT, USA), and the core and cladding at the end of the fibers were then cut using a precision fiber cleaver (Fujikura, model CT-30, Vista, CA, USA).

The xerogel films were prepared using the sol–gel process at pH 10 from mixtures of phenyltriethoxysilane (PhTEOS) and tetraethoxysilane (TEOS). Absolute ethanol and aqueous ammonia were supplied by Merck (Darmstadt, Germany). Silicon precursors with purity greater than 98% were obtained from Sigma-Aldrich (Steinheim, Germany). All chemicals were used without further purification. Water was deionized and purified with a Milli-Q system (model 185, Millipore, Mosheim, France). For the preparation of the xerogel films at pH 10, a mixture of PhTEOS and TEOS was first mixed with ethanol, then water was added dropwise. The molar ratio of PhTEOS/TEOS/ethanol/water was x:(1−x):6:6, respectively. Three mixtures of PhTEOS-TEOS were prepared with x equal to 0.3, 0.4 and 0.5. After adjusting the pH to 10.0 by addition of 0.5 M NH_3(aq)_, the samples were placed on a shaker inside an oven at 333 K (Hotcold A, Selecta, Barcelona, Spain) for two hours.

The deposition of films on the optical fibers was performed by dip coating. After two hours, colloidal suspensions were withdrawn from the oven and allowed to cool at room temperature. The tip of the fibers was dipped into the corresponding sol and then pulled out at a constant speed of 8.3 cm min^−1^. Two replicates were prepared for each colloidal suspension. The films were dried for two days under atmospheric pressure at 296 ± 2 K to create a layer of xerogel. The remaining sols were again placed in the oven and left to gel. Five milliliters of ethanol were added to the alcogels to favor aging for one week. The samples were dried under atmospheric conditions. In the current study, the samples are referred to as Ph30, Ph40 and Ph50, where the number represents the molar percentage of PhTEOS in the mixture of siliceous precursors.

### Sample characterization

Fourier transform infrared (FTIR) spectra were obtained using a Nicolet Avatar 360 FTIR spectrometer (Madison, USA). For each sample, 32 scans in the 4000–400 cm^−1^ spectral range were recorded with a resolution of 4 cm^−1^. The KBr pressed-disk technique was used at two sample concentrations: 0.6 mg was dispersed in 199.4 mg of KBr to observe the details of the recorded spectra in the 2200–400 cm^−1^ region, and 2.0 mg was dispersed in 198 mg of KBr to analyze the 4000–2200 cm^−1^ region. The pellets were heated in a furnace overnight at 423 K to minimize the amount of water adsorbed by the samples.

X-ray diffraction (XRD) patterns were acquired at ambient temperature on a Siemens D-500 X-ray diffractometer with a copper rotating anode and a graphite monochromator to select the Cu Kα_1,2_ wavelength. The device was operated at 40 kV and 80 mA. The measurements were taken in the step-scanned mode from 5° ≤ 2θ ≤ 80° in steps of 0.03°, with a counting rate of 1 s step^−1^.

Nitrogen adsorption at 77 K was performed using an ASAP 2010 volumetric adsorption analyzer (Micromeritics, Norcross, GA, USA). 100 mg of each sample were accurately weighed out in an elongated Pyrex glass tube. Before the adsorption analysis, the samples were degassed for at least 12 h at 423 K at the degassing port of the adsorption apparatus, with a residual vacuum of 0.70 Pa. The specific surface area of the silica xerogels was calculated from the nitrogen adsorption data using the Brunauer–Emmett–Teller (BET) method in a relative pressure range of 0.05–0.30, according to the criteria described by Rouquerol et al. [17–18]. The micropore volume and characteristic energy were obtained by the Dubinin–Radushkevich method [19]. The volume of the pores was calculated using 0.808 g cm^−3^ as the liquid density of N_2_ at 77 K. The pore size distributions were obtained from the N_2_ adsorption data by applying the BJH method [17].

## Experimental

The measuring device comprised an optical system, a measuring cell, a vacuum and dosing system, controllers for temperature and pressure inside the measuring cell, and software for programing the response cycles and storing experimental data. The optical system included a 50/50 coupler with a 62.5 µm core diameter connected to a white light source (DH-2000, Mikropack), a fiber optic sensing element operated in reflection mode, and a spectrometer (USB 4000, Ocean Optics). An automaton controlled the electrovalves, pressure and temperature probes, vacuum pump, and heating bath. After evacuating the measuring chamber to less than 2 hPa for 120 s, the pressure was increased to a predetermined value that was maintained for 5 s, and the gas-tight chamber was again evacuated to achieve the initial pressure, which was maintained at 0 ± 2 hPa for 120 s. Eight pressure values were included in each cycle, whose randomized values were 10, 40, 20, 50, 30, 5, 60 and 70 hPa. Four cycles were registered in each run. To assess the isosteric adsorption enthalpy, we registered the response at 288, 298, 308, and 323 K. For each temperature, the maximum pressure was 70 hPa to avoid condensation of the analyte on the film, measuring cell, or in the dosing tubes.

The exposure of the xerogel film to the analyte vapor inside the measuring cell changes the reflected signal, which is bifurcated back to the coupler and measured by the spectrometer. The response was as follows:

[2]
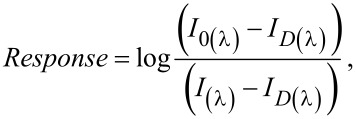


where *I*_0(λ)_ is the intensity of the reference signal, *I*_D(λ)_ is the intensity of the dark reference signal, and *I*_(λ)_ is the intensity received from the sample; all intensities were obtained by integrating the signal between 500 and 700 nm. The dark and reference intensities were first registered with the sensing element inside the evacuated measuring chamber. Working under static volumetric conditions and assuming ideal behavior, the analyte vapor concentration (*C*) on the measuring cell is related to the vapor pressure by the following equation:

[3]
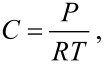


where *R* is the gas constant, *P* is the pressure, and *T* is the temperature.

## Results and Discussion

### Materials characterization

**Figure 1:**
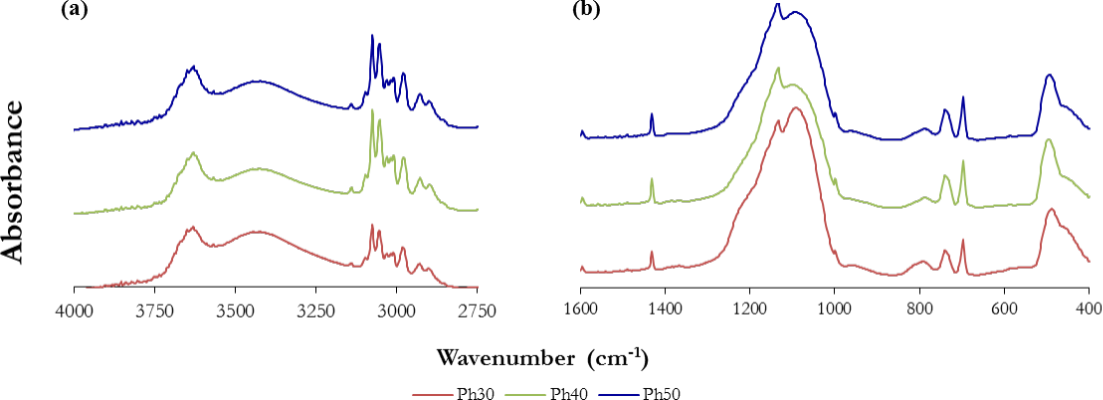
Infrared spectra of xerogels in two wavenumber ranges: (a) 4000–2750 cm^−1^ and (b) 1600–400 cm^−1^.

Figure 1 shows the FTIR spectra of the xerogels synthesized from PhTEOS/TEOS mixtures with 30, 40 and 50% PhTEOS in the mixture of the silica precursors, including two wavenumber ranges: (a) 4000–2750 cm^−1^ and (b) 1600–400 cm^−1^. The 2750–1600 cm^−1^ range was not included because of the lack of relevant bands. The incorporation of the phenyl groups into the xerogels can be monitored by the peaks located between 3100 and 3000 cm^−1^, which are assigned to C–H vibrations of the aromatic ring. The peaks at ≈3055 cm^−1^ and 3076 cm^−1^ are attributed to the C–H vibrations of the phenyl groups. The peak at ≈1431 cm^−1^ is attributed to the C=C vibration of the aromatic ring [20], and the peaks at 739 and 698 cm^−1^ are distinctive of benzene [21].

The three xerogels have the most intense bands at approximately 1090 cm^−1^ and 1132 cm^−1^. These arise from the asymmetric vibration of the siloxane bonds constituting the skeletal SiO_2_ network [22–23] and from the octameric cages induced by the phenyl groups [24] , respectively. The band at ≈3650 cm^−1^ is due to isolated silanol bonds [22]. The absorbance of the 3650 cm^−1^ band is similar for the three xerogels, which confirms that polar groups are present in the xerogels and that the surface is heterogeneous.

**Figure 2:**
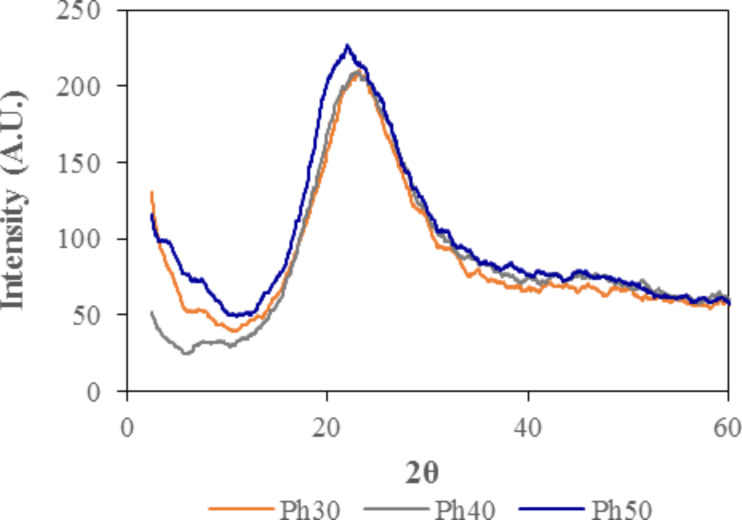
X-ray diffraction patterns of the xerogels synthesized from PhTEOS/TEOS mixtures with 30% (Ph30), 40% (Ph40) and 50% (Ph50) PhTEOS in the mixture of silica precursors.

The X-ray diffractograms of the three xerogels are shown in Figure 2. The three diffractograms have a wide peak at 2θ angles between 11° and 35°, which is characteristic of amorphous silica. The maximum of the peak decreased with increasing the percentage or PhTEOS. This band is associated with the siloxane bonds. In particular, the decrease in the maximum of the angle 2θ is related to the elongation of the silanol bond due to the inductive and steric effects of phenyl groups.

**Figure 3:**
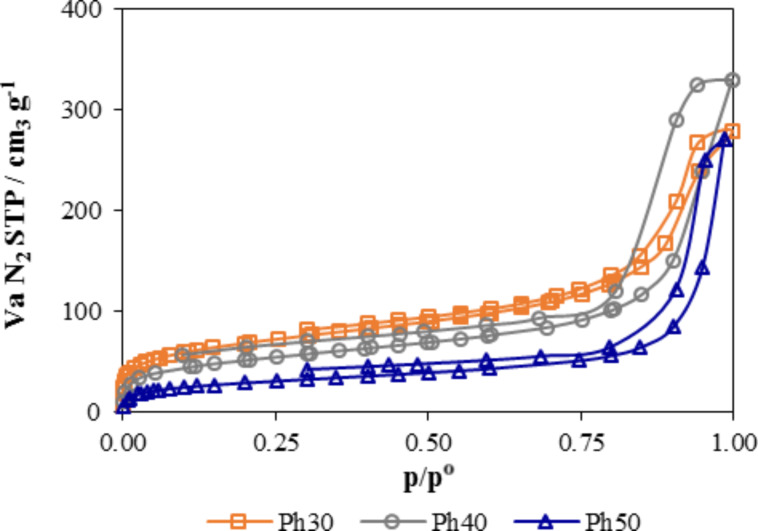
Adsorption–desorption isotherms of N_2_ at 77 K.

The N_2_ adsorption–desorption isotherms at 77 K are shown in Figure 3. The three isotherms belong to type IV with hysteresis loops of type H-2, according to the International Union of Pure and Applied Chemistry classification [17]. The Type IV isotherms are characteristic of mesoporous materials common in many inorganic oxide gels having interconnected pore networks. The amount of adsorbed N_2_ decreased with an increase in the molar percentage of PhTEOS in the mixture of silicon precursors. As the percentage of PhTEOS increased, the position of the capillary condensation step shifted towards higher relative pressure, indicating an increase in pore size. The hysteresis loops did not close at p/pº of 0.42, as is common in most N_2_ isotherms. This phenomenon is related to the irreversible adsorption in pores with opening diameters close to the kinetic diameter of the adsorbate [25]. For the PhTEOS hybrid xerogels, the non-closure of the hysteresis loop could be associated with the presence of cage-like domains in the xerogels.

The incorporation of organic groups in the xerogel framework affected the porous texture of the xerogels. Organic groups reduce the connectivity of Si atoms in the polymeric network from four to three. They also reduced size and volume of pores. The textural parameters of the xerogel deduced from the nitrogen adsorption data at 77 K are summarized in Table 1. The xerogels had specific surface areas that ranged from 242 m^2^ g^−1^ for Ph30 to 103 m^2^ g^−1^ for Ph50. The characteristic energy, expressed as kJ mol^−1^, was 15.9 for 30% PhTEOS, 13.5 for 40% PhTEOS, and 10.4 for 50% PhTEOS. Therefore, the adsorbent–nitrogen interaction decreased with the increasing percentage of the hybrid silicon precursor.

**Table 1:** Textural parameters of the hybrid xerogels: BET specific surface area, *a*_s(BET)_; total pore volume, *V*_total_; micropore volume, *V*_micro_; average pore size of the mesopores, APS_meso_; and characteristic energy, *E*_a_.

Parameter	Ph30	Ph40	Ph50

*a*_s(BET)_ (m^2^ g^−1^)	242 ± 2	183 ± 2	103 ± 1
*V*_total_ (cm^3^ g^−1^)^a^	0.430	0.511	0.419
*V*_micro_ (cm^3^ g^−1^)^b^	0.042	0.026	0.014
APS_meso_ (nm)^c^	7.45	15.3	9.02
*E*_a_ (kJ mol^−1^)^b^	15.9	13.5	10.4

^a^Total pore volumes were obtained from N_2_ adsorption at p/pº = 0.99. ^b^Micropore volumes and characteristic energies were obtained by the Dubinin–Radushkevich method. ^c^Pore size distributions were obtained from N_2_ adsorption data by applying the BJH method.

### Time-response curves

**Figure 4:**
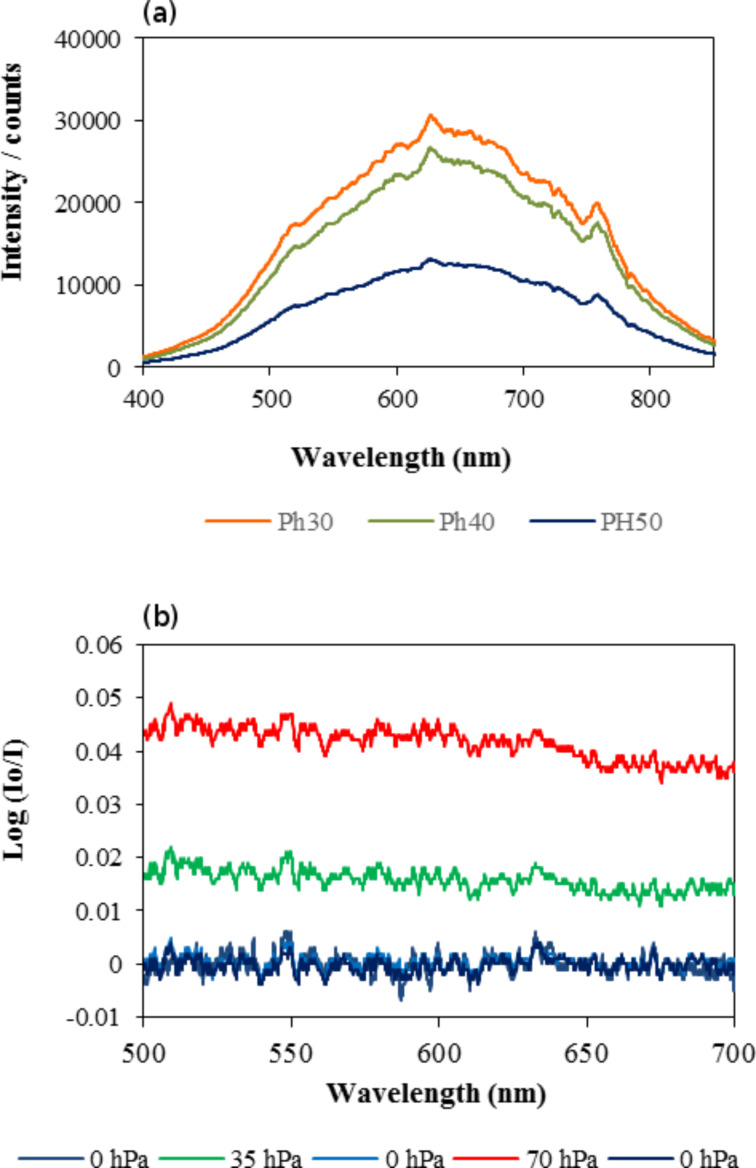
(a) Intensity of the reflected light by sensing elements Ph30, Ph40, and Ph50 at 298 K under vacuum conditions. (b) Response of the sensing element Ph40 as a function of wavelength at 298 K under vacuum and after dosing *n*-hexane to reach 35 and 70 hPa.

Figure 4a includes the raw spectra of the as-fabricated sensing elements obtained under vacuum, in which the intensity of the reflected light by sensing elements Ph30, Ph40 and Ph50 at 298 K is plotted as a function of the wavelength. The features of the spectra are those of the radiation of the white light source. Figure 4b shows the response of the sensing element Ph40 as a function of wavelength at 298 K under vacuum, and after dosing *n*-hexane to reach 35 and 70 hPa. The response is the logarithm of the ratio of the initial intensity with the chamber evacuated (*I*_0_) to the intensity in presence of *n*-hexane. The log (*I*_0_/*I*) result significantly increased above the baseline when *n*-hexane was dosed and the response went back to the baseline when the chamber was degassed to 0 ± 2 hPa. For subsequent experiments, time–response curves were obtained by integrating the signal between 500 and 700 nm.

**Figure 5:**
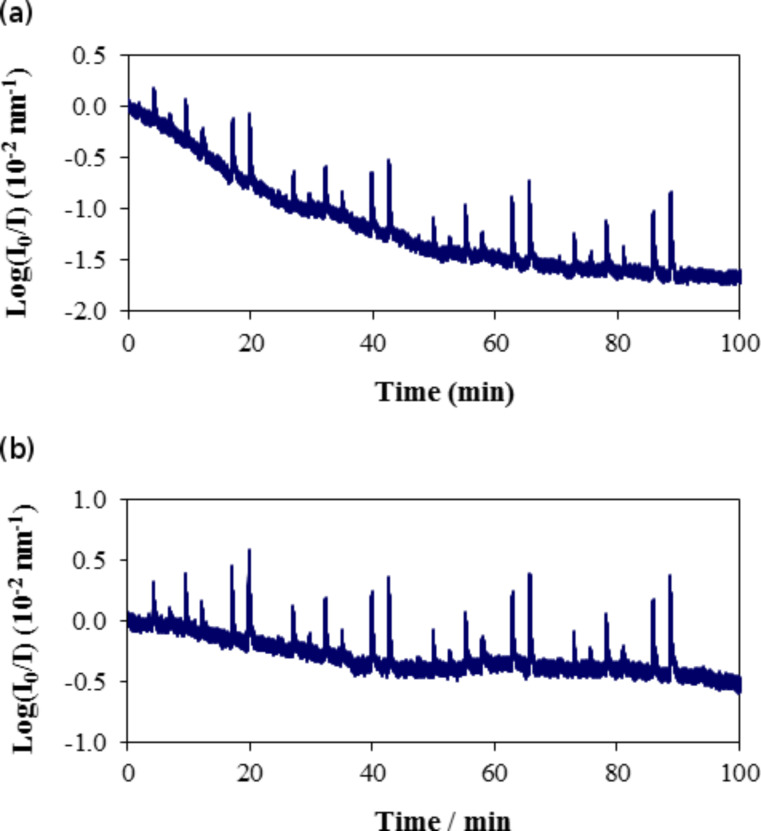
Conditioning of the sensing element Ph40 in the presence of *n*-hexane at 323 K: (a) first and (b) second runs of four cycles.

The sensing elements that include a film of xerogel at the tip of an optical fiber required conditioning to stabilize the baseline and to enlarge the response. As an example, Figure 5 shows the response of the sensing element Ph40 in the presence of *n*-hexane at 323 K. The assay included four cycles of eight prefixed pressure values that were randomized to minimize spurious effects due to sequential increases or decreases in the analyte concentration. The response is the logarithm of the ratio of the initial intensity with the chamber evacuated (*I*_o_) to the intensity as a function of time (*I*) normalized to the integration range. The baseline decreased by approximately 3.3 units from the beginning of the measurements, which is almost twice the maximum response in the presence of 70 hPa of *n*-hexane. The slope of log (*I*_o_/*I*) as a function of time was 2.9 × 10^−3^ s^−1^ for the first cycle and 4.0 × 10^−5^ s^−1^ for the fourth cycle.

**Figure 6:**
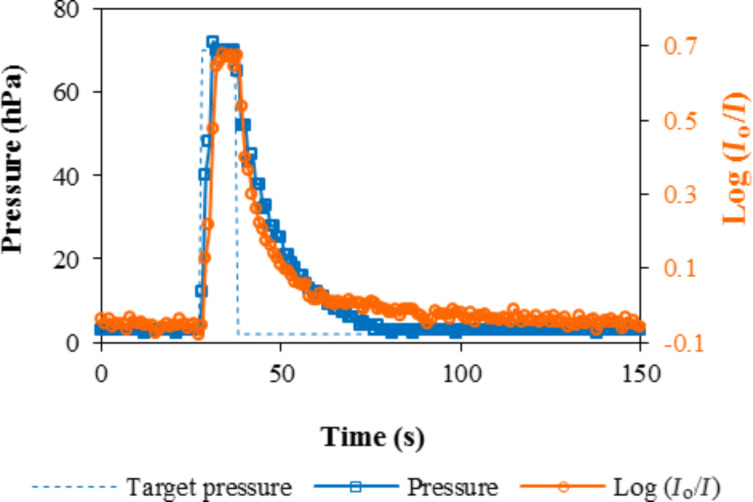
Detail of the variation of pressure and the response of the Ph40 sensing element in the presence of 70 hPa of *n*-hexane.

Figure 6 shows detail of the variation of pressure and the response of the Ph40 sensing element in the presence of 70 hPa of *n*-hexane after conditioning. The plot includes the target pressure (dashed blue line) and the actual pressure (continuous blue line) on the main y-axis; the response is plotted on the secondary y-axis. The response was synchronized with the variation of *n*-hexane inside the measuring chamber. The target pressure for dosing was achieved in less than 3 s. Degassing required 46 s and was limited by the volume of the measuring chamber, the dead volume of the system, and the performance of the vacuum pump.

**Figure 7:**
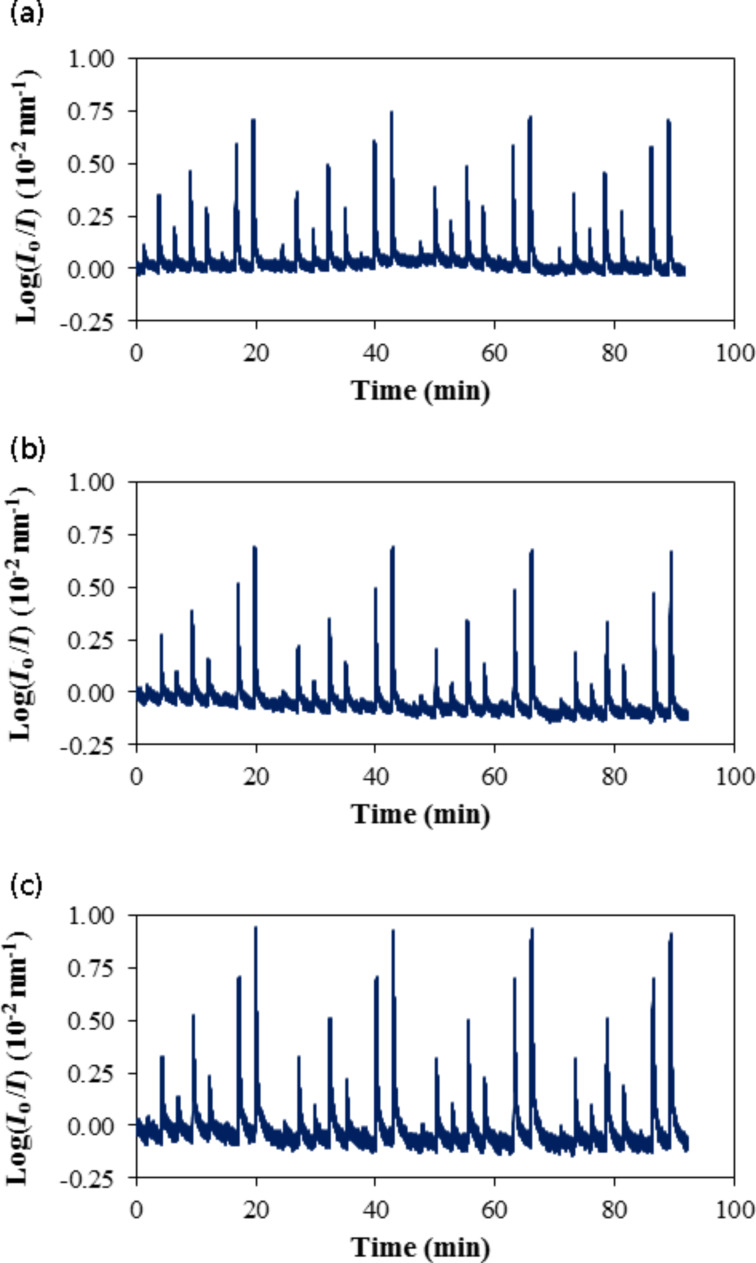
Time–response curves for three sensing elements in the presence of *n*-hexane at 298 K: (a) Ph30, (b) Ph40 and (c) Ph50.

The three sensing elements prepared from mixtures of PhTEOS–TEOS containing 30%, 40% and 50% PhTEOS in the mixture of their silica precursors responded to the presence of *n*-hexane. As an example, Figure 7 includes the time–response curves for the three sensing elements in the presence of *n*-hexane at 298 K. The signal was obtained by integrating the reflected radiation in the range of 500–700 nm, expressed as log(*I*_o_/*I*). The noise was similar for the three sensing elements. The target pressure at each step was reached at between one second for 5 and 10 hPa and 7 s for 70 hPa. The sensing element response in the presence of *n*-hexane can be characterized by a fast increase in log(*I*_o_/*I*) due to a pressure increase, followed by an exponential decrease to a steady-state value of the signal upon evacuating the measuring chamber for 180 s. The baseline was stable, and the response of the sensing element was reproducible and, in general, varied from 2% for intermediate pressure values and 6% for lower and upper pressure values.

**Figure 8:**
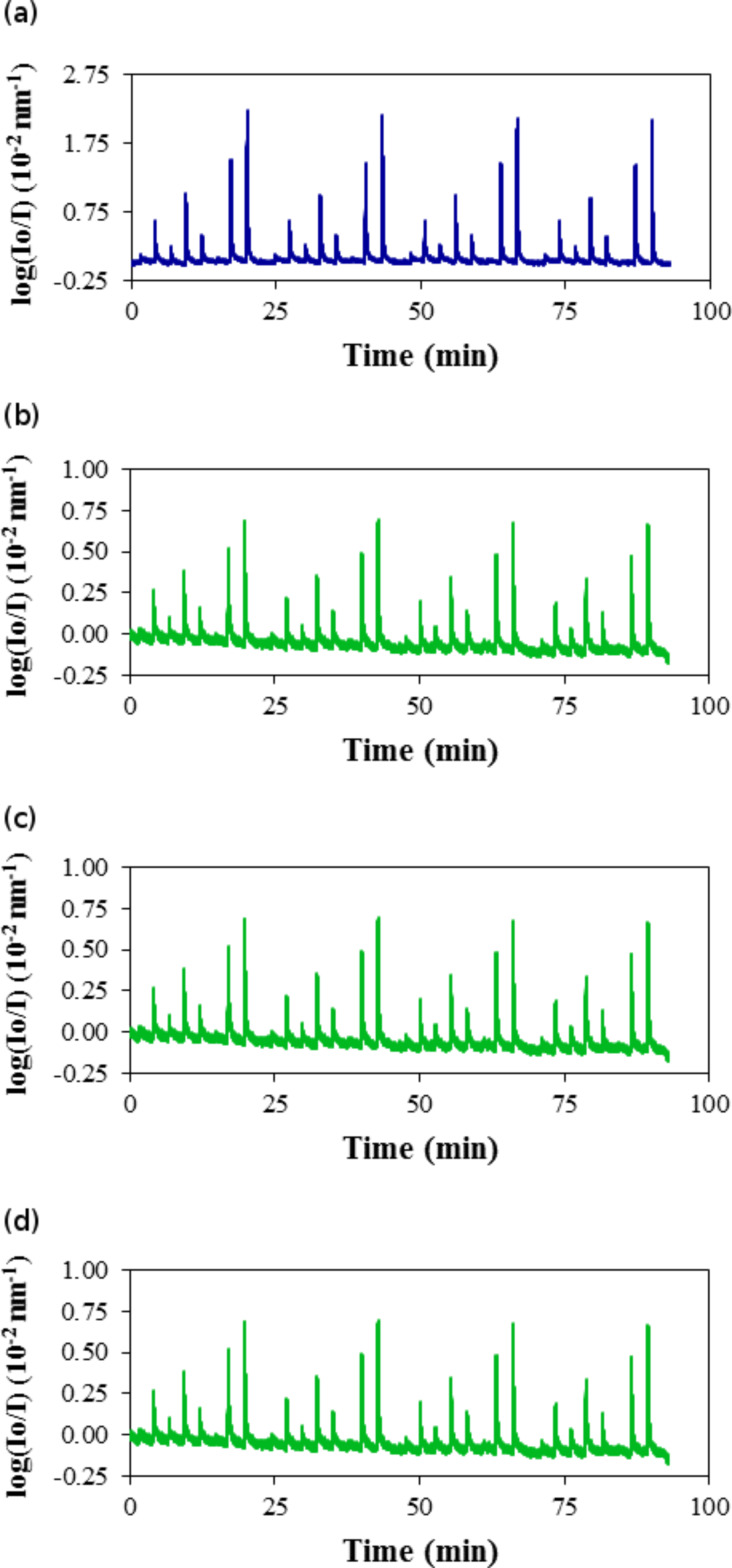
Time–response curves for the sensing element Ph40 in the presence of *n*-hexane: (a) 288 K, (b) 298 K, (c) 308 K and (d) 323 K.

The response of each sensing element decreased with increasing temperature. Figure 8 shows a comparison of the time–response curves for the sensing film Ph40 in the presence of *n*-hexane at 288, 298, 308 and 323 K. As the temperature increased, the response at a given pressure of *n*-hexane decreased, which was due to the decrease in the amount of adsorbed *n*-hexane with temperature. The reflectance decreased with temperature; for example, at a pressure of 70 hPa, the normalized signal was 2.1 at 288, 0.80 at 298 K, 0.42 at 308 K, and 0.22 at 323 K.

**Figure 9:**
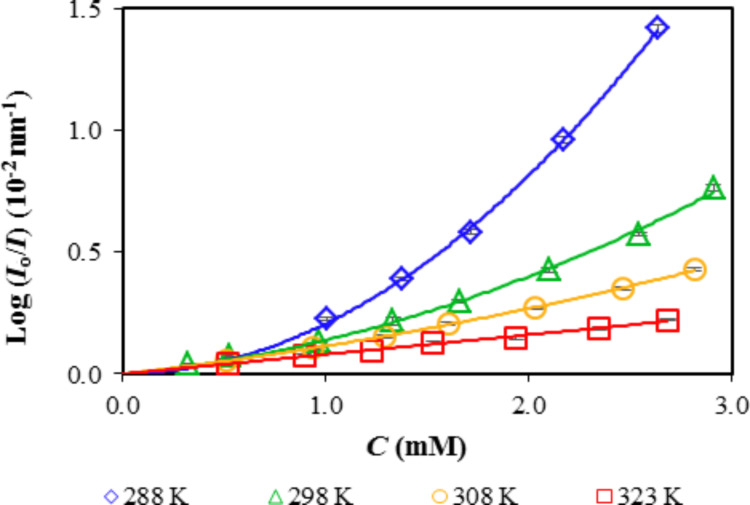
Calibration curves for the Ph40 sensing element in the presence of *n*-hexane at 288, 298, 308, and 323 K.

These time–response curves provided the data for drawing the calibration curves, in which the response was plotted as a function of the vapor concentration, considering that vapors behave as ideal gases. From the four experimental data points at each pressure, we obtained the mean, standard deviation, and coefficient of variation. Figure 9 shows the calibration curves for the Ph40 sensing element in the presence of *n*-hexane at 288, 298, 308, and 323 K. The curves depict the variation of the reflectance on a logarithmic scale as a function of concentration. The experimental data were fitted to a second-degree polynomial function using Excel software under the restriction of *C* = 0.

Table 2 includes the analytical parameters for the Ph30, Ph40 and Ph50 sensing elements in the presence of *n*-hexane deduced from the time–response curves at 288, 298, 308, and 323 K. The table includes the second- and first-order coefficients, quadratic regression coefficients, coefficients of variation, and limits of detection. The second-order coefficients exponentially decreased with increasing temperature for the three sensing elements, which indicates that the calibration curves became linear with temperature. This behavior appeared to be related to the adsorption of *n*-hexane on the films, which is an exothermic process, and to the relative pressure of the analyte, which decreased with temperature because the pressure values were the same in each run. Except for the sensing element Ph50 at 323 K, the quadratic regression coefficients were above 0.99, which reflects the agreement between the experimental data and the model. In general, the coefficients of variation were smaller at 288 and 298 K than at 323 K. We applied a two-factor analysis of variance (ANOVA), which included the temperature and the sensing element as source of variation, to the first- and second-order coefficients that describe the calibration curves. The null hypothesis could only be rejected for the effect of temperature on the second-order coefficient. Therefore, the response of the sensing elements significantly decreased with the temperature.

**Table 2:** Analytical parameters for the Ph30, Ph40, and P50 sensing elements in the presence of *n*-hexane deduced from the time-response curves at 288, 298, 308, and 323 K, including the second- (a) and first-order (b) coefficients, quadratic regression coefficients (R^2^), coefficients of variation (COV) and limits of detection (LoD).

*T*	*a*	*b*	R^2^	COV	LoD	
(K)	(10^−2^ nm^−2^ mM^−2^)	(10^−2^ nm^−1^ mM^−1^)		(%)	(mM)	(hPa)

**Ph30**						
288	0.100 ± 0.084	0.187 ± 0.028	0.993	1.11–4.7	0.269	6.43
298	0.020 ± 0.002	0.169 ± 0.007	0.999	0.99–3.99	0.309	7.02
308	0.011 ± 0.002	0.120 ± 0.007	1.000	1.23–7.95	0.428	10.9
323	−0.002 ± 0.002	0.084 ± 0.006	0.998	1.73–8.09	0.729	19.6
**Ph40**						
288	0.237 ± 0.012	−0.058 ± 0.004	0.999	0.21–5.32	0.573	13.7
298	0.061 ± 0.004	0.078 ± 0.012	0.997	1.05–7.88	0.418	10.4
308	0.020 ± 0.002	0.094 ± 0.006	0.999	0.94–9.04	0.406	10.4
323	0.000 ± 0.003	0.080 ± 0.010	0.994	1.29–7.08	0.703	18.8
**Ph50**						
288	0.191 ± 0.09	0.172 ± 0.027	0.998	0.93–8.45	0.355	8.5
298	0.070 ± 0.004	0.155 ± 0.011	0.997	0.977–6.69	0.457	11.3
308	0.022 ± 0.004	0.111 ± 0.013	0.995	2.65–6.14	0.631	14.3
323	−0.001 ± 0.014	0.110 ± 0.019	0.985	2.32–7.28	0.678	18.2

The coefficient of variation (COV) directly relates to the dispersion of the results. The largest COV was for the response measured at 5 and 10 hPa. The limit of detection (LoD) was calculated by the propagation of errors approach, which includes the variability of the blank measurements and the uncertainty of the sample measurements.

[4]
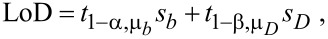


where *t*_1−α,µb_ stands for the t-student of the blank, *s*_b_ is the standard deviation of the blank, *t*_1-β, µD_ is the t-student of the sample and *s*_D_ is the standard deviation of the sample. The standard deviation of the noise was estimated by averaging 10 experimental data points from the baseline, and the standard deviation of the measurement was obtained by measuring the response in the presence of 20 hPa of *n*-hexane. For a confidence level of 95%, coefficient *t*_0.95;9_ was 1.83, and *t*_0.95; 3_ was 2.35. The limits of detection varied from 0.269 mM for Ph30 at 288 K to 0.729 for the same sensing element at 323 K, which corresponded to 6.4 hPa at 288 K, and 19.6 hPa at 323 K in pressure units.

### Isosteric enthalpy of adsorption

The experimental procedure for determining the isosteric enthalpy of adsorption in the ideal gas approximation consists of plotting the response of the sensing element, log(*I*_o_/*I*), as a function of the equilibrium pressure or its equivalent equilibrium concentration (*C* = *P*/(*RT*)) obtained at several fixed temperatures (Figure 9). A constant fixed response or isostere is chosen to find the equilibrium *T*–*C* values. The effect of temperature on the response was investigated at four temperatures between 288 and 323 K. As we have explained in the Introduction section, when the analyte molecules are adsorbed on the silica xerogel the reflected intensity varies. Adsorption is an exothermic process, in which when a vapor molecules is adsorbed on a surface loses translational entropy because it changes from tree to two degrees of freedom [15,17]. When the two phases are at equilibrium, their chemical potential must be equal:

[5]
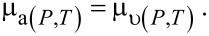


For a closed system (*V* = constant) without expansion work and a constant composition for each phase,

[6]
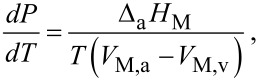


where Δ_a_*H*_M_ is the isosteric enthalpy of adsorption and *V*_M,a_ and *V*_M,v_ are the molar volumes of the adsorbed and vapor phases, respectively. Assuming that the vapor behaves as an ideal gas at low pressure,

[7]
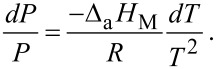


If we assume that the isosteric enthalpy of adsorption is independent of temperature, which is true if Δ*T* is not too large, this equation integrates into

[8]
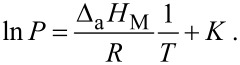


**Figure 10:**
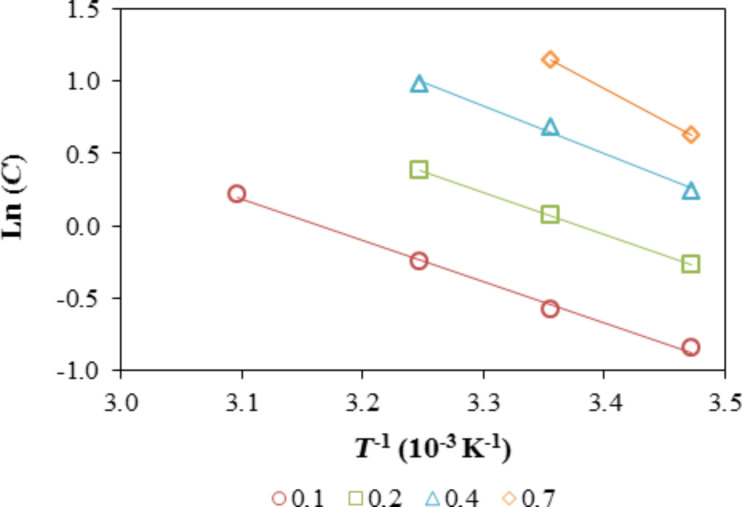
Clausius–Clapeyron plots, ln *C* plotted against the reciprocal absolute temperature for the response of the Ph30 film in the presence of *n*-hexane for log(*I*_o_/*I*) = 0.1, 0.2, 0.4, and 0.7.

The plot of ln *P* or ln *C* as a function of *T*^−1^ is used to find the slope, which is related to the isosteric enthalpy of adsorption. The response of the sensing element is a function of the pressure and concentration of the analyte inside the measuring chamber; therefore, the adsorption enthalpies (Δ_a_*H*_M_) were obtained from the slope of the plots of ln *C* versus *T*^−1^. As an example, Figure 10 plots ln *C* as a function of the reciprocal temperature for the sensing element Ph30 in the presence of *n*-hexane. The slope of the plots increases with the relative response.

**Figure 11:**
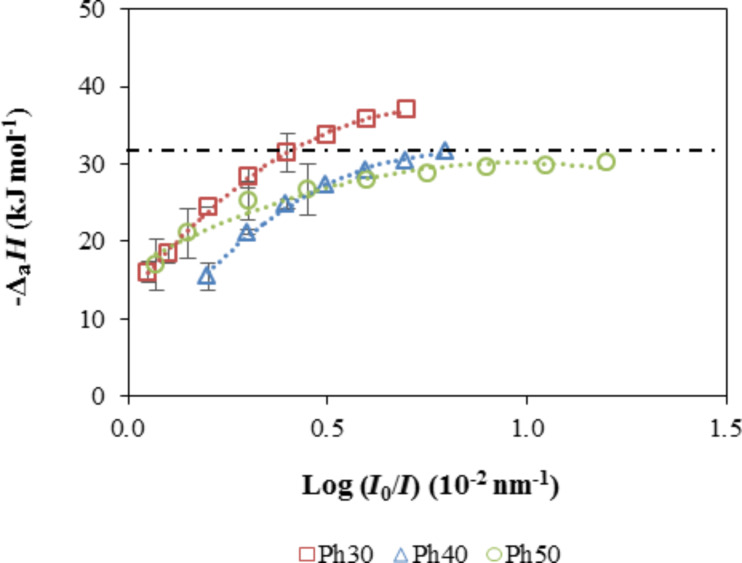
Isosteric adsorption enthalpy. Dashed line: the condensation enthalpy of *n*-hexane on a flat liquid surface.

Figure 11 shows the variation of the isosteric enthalpy of adsorption of *n*-hexane with the relative response to the maximum value for the three sensing elements. The magnitude of the isosteric enthalpy of *n*-hexane increased with the relative response and reached a plateau that stabilized at approximately −31 kJ mol^−1^ for Ph40 and Ph50 and at approximately −37 kJ mol^−1^ for Ph30. At low adsorption, the adsorbate–adsorbent interaction is dominant [26]. Because *n*-hexane is a non-polar compound, it exhibits almost non-specific interactions with the surface of the hybrid xerogels. The enthalpy of adsorption for *n*-hexane for Ph40 and Ph50 was similar to the molar enthalpy of condensation of the adsorbate on a flat liquid surface (Δ_cond_*H* = −31.8 kJ mol^−1^), which suggests that the adsorbate condenses in the pores of the films. For the Ph30 xerogel, the adsorption enthalpy differed from the enthalpy of condensation by ≈7 kJ mol^−1^, which can be due to confinement effects related to the condensation of adsorbates in narrower mesopores [27–28]. The solid–fluid interaction increases as the pore size diminishes and, therefore, the adsorption enthalpy in the phase transition region [26].

## Conclusion

We investigated the response, in the presence of *n*-hexane, of three sensing elements prepared from mixtures of PhTEOS–TEOS containing 30, 40 and 50% PhTEOS in silica precursor mixture. The incorporation of organic groups into the xerogel framework decreased the characteristic energy of the films. At a given pressure of *n*-hexane, the response of each sensing element decreased with temperature, denoting an exothermic process that confirms the role of adsorption in the overall performance of the sensing elements. There were significant differences in the second-order coefficients of the regression equations for the three sensing elements. The limits of detection varied from 0.269 mM for Ph30 at 288 K to 0.729 mM for the same sensing element at 323 K, which corresponded to 6.4 hPa at 288 and 19.6 hPa at 323 K in pressure units. The isosteric adsorption enthalpies were obtained from the calibration curves at different temperatures. The magnitude of the isosteric enthalpy of *n*-hexane increased with the relative response and reached a plateau that stabilized at approximately −31 kJ mol^−1^ for Ph40 and Ph50 and at approximately −37 kJ mol^−1^ for Ph30, which indicates that the adsorbate–adsorbent interaction was dominant at lower relative pressure and condensation of the adsorbate on the mesopores was dominant at higher relative pressure.

## References

[1] Elosua C, Matias I R, Bariain C, Arregui F J (2006). Sensors.

[2] McDonagh C, Burke C S, MacCraith B D (2008). Chem Rev.

[3] Wang X-D, Wolfbeis O S (2013). Anal Chem.

[4] Wang X-D, Wolfbeis O S (2016). Anal Chem.

[5] Melissinaki V, Farsari M, Pissadakis S (2015). IEEE J Sel Top Quantum Electron.

[6] Melissinaki V, Konidakis I, Farsari M, Pissadakis S (2016). IEEE Sens J.

[7] Born M, Wolf E (2005). Principles of optics. Electromagnetic theory of propagation, interference and diffraction of light.

[8] Pisco M, Consales M, Campopiano S, Viter R, Smyntyna V, Giordano M, Cusano A (2006). J Lightwave Technol.

[9] Consales M, Crescitelli A, Penza M, Aversa P, Veneri P D, Giordano M, Cusano A (2009). Sens Actuators, B.

[10] Abdelghani A, Chovelon J M, Jaffrezic-Renault N, Lacroix M, Gagnaire H, Veillas C, Berkova B, Chomat M, Matejec V (1997). Sens Actuators, B.

[11] Abdelmalek F, Chovelon J M, Lacroix M, Jaffrezic-Renault N, Matejec V (1999). Sens Actuators, B.

[12] Cherif K, Mrazek J, Hleli S, Matejec V, Abdelghani A, Chomat M, Jaffrezic-Renault N, Kasik I (2003). Sens Actuators, B.

[13] Echeverría J C, de Vicente P, Estella J, Garrido J J (2012). Talanta.

[14] Echeverría J C, Faustini M, Garrido J J (2016). Sens Actuators, B.

[15] Atkins P W (1998). Physical Chemistry.

[16] Stadie N (2013). Synthesis and thermodynamic studies of physisortive energy storage materials.

[17] Rouquerol F, Rouquerol J, Sing K S W (2014). Adsorption by powders and porous solids. Principles, methodology and applications.

[18] Brouwer S, Groen J C, Peffer L A A, Llewellyn P L, Rodriguez-Reinoso F, Rouquerol J (2007). Studies in Surface Science and Catalysis;.

[19] Estella J, Echeverría J C, Laguna M, Garrido J J (2007). J Non-Cryst Solids.

[20] Li Y-S, Wang Y, Ceesay S (2009). Spectrochim Acta, Part A.

[21] Hu Q, Li J J, Hao Z P, Li L D, Qiao S Z (2009). Chem Eng J.

[22] Innocenzi P (2003). J Non-Cryst Solids.

[23] Innocenzi P, Falcaro P, Grosso D, Babonneau F (2003). J Phys Chem B.

[24] Park E S, Ro H W, Nguyen C V, Jaffe R L, Yoon D Y (2008). Chem Mater.

[25] Zhang C, Babonneau F, Bonhomme C, Laine R M, Soles C L, Hristov H A, Yee A F (1998). J Am Chem Soc.

[26] Qiao S Z, Bhatia S K, Nicholson D (2004). Langmuir.

[27] Jänchen J, Stach H, Busio M, van Wolput J H M C (1998). Thermochim Acta.

[28] Tanchoux N, Trens P, Maldonado D, Di Renzo F, Fajula F (2004). Colloids Surf, A.

